# Cell fusion in osteoclastogenesis

**DOI:** 10.1042/BST20253131

**Published:** 2025-12-19

**Authors:** Leonid V. Chernomordik, Kamran Melikov

**Affiliations:** 1 Section on Membrane Biology, Eunice Kennedy Shriver National Institute of Child Health and Human Development, National Institutes of Health, Bethesda, MD, 20892, U.S.A.

**Keywords:** Annexin A5, fusion pathway, osteoclast fusion, phosphatidylserine, Syncytin 1

## Abstract

Multinucleated osteoclasts, generated by fusion of mononucleated precursors, play an essential role in the lifelong remodeling of our bones. Since within the physiological range of osteoclast sizes, their bone-resorbing activity grows with successive fusion events, both initiation of this fusion reaction and its switch off are tightly controlled. In this review, we discuss the mechanisms and proteins that facilitate and regulate this fusion process. The pathway of membrane rearrangements in osteoclast fusion shares many mechanistic motifs with other physiologically important cell–cell fusion processes, such as the formation of multinucleated skeletal muscle cells. However, the protein machinery involved in catalyzing these rearrangements in osteoclasts is still poorly understood. A better understanding of the mechanism of osteoclast fusion will hopefully lead to more effective approaches for treating skeletal diseases caused by excessive or insufficient bone resorption.

## Introduction

Lifelong maintenance and repair of our bones is a dynamic process based on the tightly co-ordinated formation of new bone by osteoblasts and degradation of old and damaged bone by osteoclasts [1]. Osteoblasts, the only bone-forming cells in vertebrates, originate from stromal cells in the bone marrow, chondrocytes in the growth plate, and some other cell types [2]. Osteoclasts, the only cells that resorb mineralized bone matrix, are terminally differentiated cells of monocyte-macrophage lineage [3]. An imbalance between the activities of these cell types leads to various skeletal diseases, including osteoporosis—a metabolic bone disorder affecting millions of people worldwide. Osteoporosis occurs when excessive osteoclast activity decreases bone density and increases risk of fractures [4,5].

Osteoclasts are derived through a series of complex, several-day-long differentiation processes that are dependent on two cytokines produced primarily by cells of osteoblast lineage - osteoblasts and osteocytes [6]. First, macrophage colony-stimulating factor (M-CSF) stimulates proliferation of monocytes. Then binding of the receptor activator for NF-κB (nuclear factor κB) ligand (RANKL) to its receptor, RANK, at the surface of the cells activates the Nuclear Factor of Activated T-cells cytoplasmic 1 (NFATc1)-dependent transcriptional program that controls the formation of mononucleated osteoclast precursors. The fusion of these precursors generates mature multinucleated osteoclasts, which then spread on the bone surface, form the sealing zone loaded with protons and proteases, and begin dissolving the mineral and organic components of the bone.

Typically, human osteoclasts in healthy tissue have approx. three to ten nuclei [7] and, thus, regardless of the sequence of fusion events, including fusions between mononucleated and multinucleated osteoclasts and between multinucleate osteoclasts, are generated by two to nine cell fusion events [8]. Since larger osteoclasts, generated by a larger number of cell fusion events, normally have a higher bone-resorbing activity, fusion efficiency plays an important role in the regulation of osteoclast function [7]. While microscopy analysis shows that each osteoclast fusion event takes only seconds to minutes, the cell fusion stage in osteoclast formation from the first to the last fusion event lasts several days. Osteoclast fusion must be tightly regulated to both prevent osteoclast fusion with unrelated cells and to switch off fusion activity when the size of osteoclasts (multinucleation) reaches a physiological range. While excessive osteoclast fusion that leads to an overabundance of normally sized or moderately larger osteoclasts results in excessive bone resorption, deregulated fusion that generates giant osteoclasts with projected areas up to a thousand-fold larger than normal reduces bone-resorbing activity of osteoclasts, leading to osteoclast-rich autosomal recessive osteopetrosis [3].

In this review, we summarize recent advancements in the research on osteoclast fusion and discuss the gaps in our understanding of this process in the context of other biological membrane fusion processes. We begin with a recap of the pathways involved in membrane rearrangements during fusion and the general mechanisms by which proteins merge membranes. We then discuss the protein machinery that regulates and mediates the formation of multinucleated cells, focusing on proteins implicated in osteoclast fusion.

### Membrane fusion pathway

At its core, membrane fusion is the merger of the lipid bilayers of the fusing membranes. Initially, cell–cell adhesion proteins, such as E-cadherins in the case of osteoclasts [9], bring the two plasma membranes (PMs) to a distance of 10–20 nm. A closer approach most likely depends on local outward PM deformations. The importance of such deformations has been emphasized by the dependence of myoblast fusion on invadosomes [10]. In another example, fusion-associated small transmembrane (FAST) proteins from reovirus, which fuse membranes, hijack host actin assembly to push FAST-carrying PM protrusions into the adjacent cell’s PM [11,12]. In addition to deformations, fusion depends on the local displacement of membrane proteins that cover membrane bilayers. The contact zone is crowded with proteins, and an opening of protein-depleted patches in the bilayers additionally crowds the proteins outside of these patches [13,14].

#### Pre-fusion bilayer contact

Equilibrium distances between protein-free lipid bilayers, corresponding to full hydration of the bilayer surfaces, depend on lipid and buffer composition and are determined by the interplay of the membrane hydration and undulations repulsion forces, on the one hand, and hydrophobic and van der Waals attraction forces, on the other hand. A closer approach is resisted by short-range but powerful hydration repulsion forces and by steric repulsions arising from the thermal motions of head groups and fluctuations in membrane thickness. These interbilayer forces and equilibrium distances between the bilayers (2–3 nm for bilayers formed from phosphatidylcholine (PC), the most abundant lipid in PM) have been characterized in depth by different groups, using different experimental approaches, including X-ray diffraction and an optical interference technique [15–18].

The research of the last decade describes an alternative type of interbilayer contacts. Several cryomicroscopy studies have reported very tight (less than 1 nm separation) and extended, sometimes micrometer-scale, contacts [19–21]. These contacts are initiated by proteins (SNAREs [20] and viral fusogens [19,21]), yet the proteins themselves are excluded from the contact zone and accumulate at its rim. Similar tight, dehydrated contacts, accompanied by lipid condensation within the contact zone, have also been observed between lipid bilayers with a large fraction of phosphatidylserine (PS) in the presence of millimolar concentration of Ca^2+^ [22]. The formation of such contacts must be driven by a strong attractive force that overcomes hydration and steric repulsions between membranes. In the case of PS bilayers in Ca^2+^, this attractive force has been proposed to originate either from bridging the PS head groups of the opposing membranes by Ca^2+^ ions [22] or to have an electrostatic origin and result from an alternating charge distribution on the surfaces of the PS membranes neutralized by in-plane adsorption of Ca^2+^ [23]. For membranes of the biologically relevant compositions in the absence of a large concentration of Ca^2+^, the origin of these attractive forces remains to be clarified. The nature of a strong attractive force that would keep the bilayers in a partially dehydrated contact at ~1 nm separation, as predicted by recent all-atom simulation studies of membrane fusion mediated by SNARE zipping- or inter-membrane lipid splitting [24], is also unclear.

#### Lipid bilayer rearrangements

Downstream of a close pre-fusion contact between lipid bilayers of two biological membranes, disparate membrane fusion processes, including those in intracellular fusion, enveloped virus entry, and cell–cell fusion, apparently converge to form the same types of lipid intermediates observed in the fusion of protein-free lipid bilayers [25]. First, the contacting monolayers of the two membranes merge to create a hemifusion stalk, while the distal monolayers of the two membranes remain separate. The stalk formation energy decreases if lipids and/or proteins in the contacting monolayers of the membranes bend these monolayers away from the cytoplasm [25]. By convention, monolayer curvature is assigned a negative sign when lipid molecules spread their acyl chains. A hemifusion stalk formed within a partially dehydrated contact is expected to have even greater negative net curvature of the monolayers than a stalk connecting bilayers at equilibrium distances of 2–3 nm. While hemifusion enables lipid mixing between membranes, mixing of the contents within membrane-enclosed volumes develops only when a hemifusion structure breaks, merging the distal monolayers and forming a nascent fusion pore. The rim of the pore has positive curvature, and the pore’s energy decreases if lipids and membrane proteins favor the spreading of lipid headgroups. Due to the opposite net curvatures of the rims of hemifusion stalk and pore, the inverted cone-shaped lipid lysophosphatidylcholine (LPC) inhibits hemifusion when present in the contacting membrane monolayers and promotes fusion pore opening when present in the distal monolayers. Finding that LPC reversibly inhibits osteoclast fusion upstream of lipid mixing [26] suggests that this fusion reaction proceeds through the fusion-through-hemifusion pathway common to other membrane fusion processes. LPC inhibition has been used to synchronize osteoclast fusion events, allowing focused investigation of the fusion process by observing fusion upon LPC removal [26].

For lipid compositions like those of the PM, the energy of the fusion intermediates in protein-free bilayers is too high for fusion to occur spontaneously [25]. Thus, proteins should provide work to lower the energy of these intermediates by inducing mechanical stresses in proximal and distal monolayers. Proteins can bend the bilayers, bring them together into energy-intensive contact, and directly interact with lipids facilitating the formation of fusion intermediates. For hemifusion and fusion to occur, the formation of fusion intermediates must release energy accumulated at the pre-fusion bilayer contact. Can dehydrated and partially dehydrated membrane contacts serve as a platform for productive membrane fusion? The formation of dehydrated contacts was previously suggested to drive formation of hemifusion intermediates [27,28] through the accumulation of the energy of hydration repulsion accompanying the drastic decrease in the intermembrane distance. The decrease in contact area resulting from hemifusion reduces the hydration energy, thereby favoring the hemifusion reaction. Importantly, a tight, dehydrated contact in these models was established by external factors pushing the membrane together, such as local membrane undulations [27] or the hydrostatic pressure applied to the membrane surface [28,29]. These factors represented the ultimate source of energy driving the hemifusion reaction. In contrast, in the recently described systems [20,21], the tight, dehydrated contacts are pinned together by proteins at the rim of the contact but apparently not supported by any pushing forces, which means that they result from a balance of direct intermembrane interactions. In this case, the small intermembrane distance must represent a force equilibrium, which precludes the membrane’s tendency to form stalk and expand the hemifusion diaphragm. Furthermore, if the area of the energetically favorable dehydrated contact becomes comparable with the total area of a fusing membrane [19–21,30], the extension of the contact area deforms the membranes and tends to decrease the intracellular volume. This may result in the buildup of intracellular pressure and the related membrane tension to a level large enough to break the bilayers in the contact zone or outside of it, hence leading to either fusion or leakage [31]. It seems unlikely that such an uncontrolled and intrinsically leaky mechanism of fusion pore formation can be utilized in biological processes.

#### Fusion pore expansion

The formation of early nanometer-sized fusion pores does not necessarily lead to the complete unification of the volumes of the two cells. The enlargement of the pores to a micrometer- and, then, cell size-sized lumen is ATP-dependent and controlled by dynamin and other intracellular proteins [26,32,33]. Intriguingly, in many cells and physiological contexts, narrow fusion connections do not fully expand and are observed as long tunneling nanotubes, i.e., F-actin-containing membranous channels between distinct cells [34]. In osteoclasts, the formation of nanotubes, and especially, thick ( ≥ 2 μm diameter) nanotubes, correlates with and is apparently mechanistically linked to the fusion of osteoclast precursors [35,36]. The late stages of cell–cell fusion, including the formation of nanotubes and the appearance and expansion of micrometer-sized fusion pores, as well as any changes in cell shapes and the organization of cell volume, must involve rearrangements of actin cytoskeleton.

### Proteins involved in osteoclast fusion

**Figure 1:**
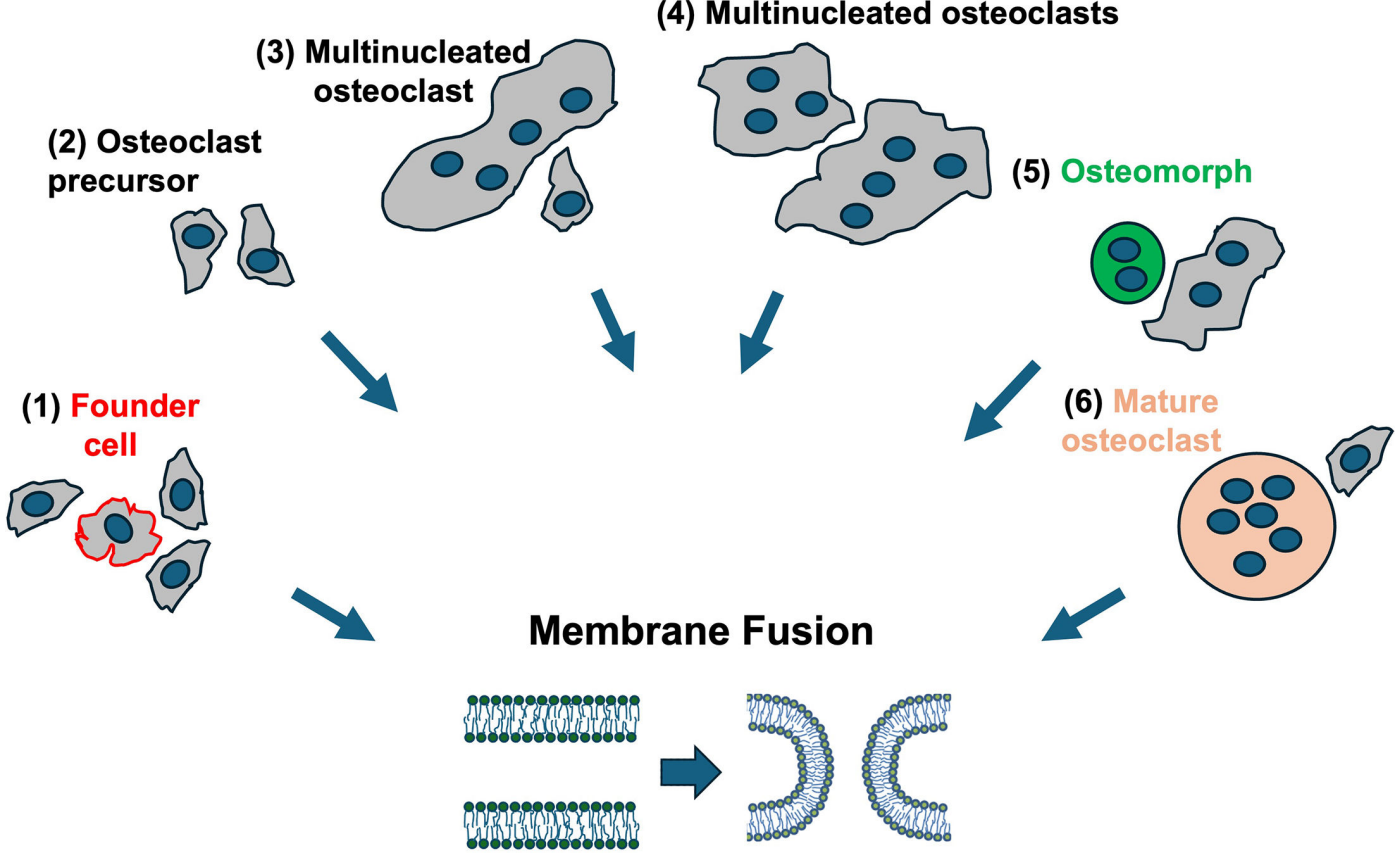
Different examples of osteoclast fusion in bone homeostasis. Fusion occurs between (1) founder and follower osteoclast precursors (2); mononucleated precursors (3); mononucleated and multinucleated osteoclasts (4); two multinucleated cells (5]; osteomorph and osteoclast; and (6) differentiating and mature bone-resorbing osteoclasts. It remains unclear how the protein machineries differ in mediating each specific type of described above.

Osteoclast fusion machinery (i) ensures that fusion happens at the right time and place in the context of cell physiology; (ii) mediates actual fusion event by bringing bilayers together and by lowering the energy barriers for fusion intermediates; and (iii) drives expansion of the nascent fusion pores to form a multinucleated cell rather than connecting two cells by a tunneling nanotube. Different combinations of these functions can be carried out by the same protein(s)/protein complexes or divided between different proteins/protein complexes that act together or independently at different stages of the fusion pathway. While we already know several proteins that the cell fusion stage of osteoclast formation depends on, this fusion is arguably among the least understood cell–cell fusion processes in the development and maintenance of mammalian tissues and organs [37]. Even the assumption that all fusion events in the formation of mature multinucleated osteoclasts are controlled by the same protein machinery and proceed via similar pathways can be an important oversimplification [38]. Osteoclasts fuse in diverse biological contexts (Figure 1). Not much is known about differences in machinery that mediates fusion between mononucleated osteoclast precursors and fusion between two multinucleated osteoclasts [38]. An additional level of complexity comes from a live-cell imaging study that divides all cells involved in osteoclast fusion into two classes: a small fraction of fusion-competent founder cells and a much larger pool of ‘fusion followers’, which fuse with founder cells but do not initiate fusion [39]. In this scenario, most osteoclast precursors in the culture of differentiating osteoclasts are expected to lack some or all of the protein components that constitute the fusion machinery. We also do not know whether any distinctions exist between protein machineries controlling the most studied fusion of monocyte-derived osteoclast precursors and those controlling other fusion events. These fusion events include fusion of relatively small osteomorphs formed by osteoclast fission back into osteoclasts [40] and fusion of mature osteoclasts during bone resorption [41].

It is thought that all osteoclast fusion processes depend on RANKL-induced multi-pass transmembrane proteins Dendritic Cell-Specific Transmembrane Protein (DC-STAMP), and osteoclast stimulatory transmembrane protein (OC-STAMP). Both proteins are indispensable for osteoclast fusion [42,43]. Despite the normal expression of osteoclast markers, DC-STAMP knockout mice and OC-STAMP knockout mice as well as ex vivo cultures from these mice incubated with M-CSF and RANKL have only mononucleated osteoclasts. Both DC-STAMP and OC-STAMP promote cell–cell fusion between osteoclast precursors when overexpressed [44], and suppressing DC-STAMP activity by antibodies inhibits synchronized osteoclast fusion uncoupled from prefusion differentiation stages [45]. However, overexpression of either or both proteins in osteoclast precursors was insufficient to mediate cell fusion in the absence of RANKL/NFATc1-dependent differentiation [46]. A large set of proteins involved in osteoclast multinucleation is found to interact with DC-STAMP and OC-STAMP and regulate their expression and/or function [44,47].

Out of a considerable number of other proteins linked to the fusion stage of osteoclastogenesis [47,48], our discussion below focuses on the proteins that seem to work together.

#### Cell-surface phosphatidylserine-associated fusion regulators

**Figure 2:**
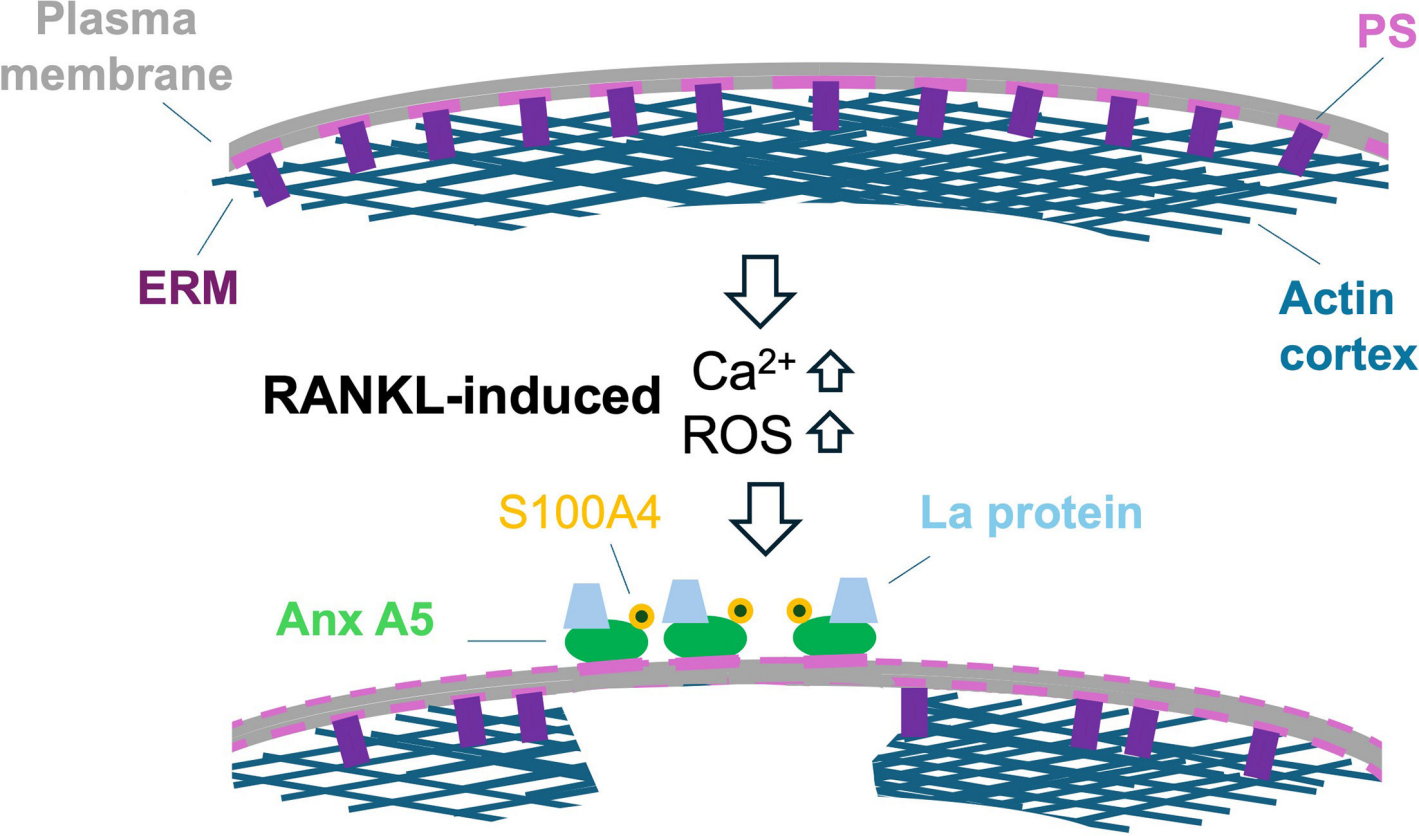
A cartoon illustrating an emerging regulatory mechanism mediated by extracellular Annexin A5 and coupling fusion of osteoclasts with several signaling pathways. Before osteoclastogenesis, PS (shown as a pink line) is almost exclusively retained in the inner leaflet of the PM. PS-bound ERM proteins (purple rectangles) link PM (gray) to the actin cortex. RANKL-RANK interactions increase intracellular Ca^2+^ and concentrations of reactive oxygen species (ROS). Ca^2+^ rise activates lipid scramblase that facilitates PS redistribution to the outer PM leaflet. Extracellular Anx A5 binds to exposed PS and further decreases PS content in the inner PM leaflet. Loss of PS in this leaflet results in a local detachment of a fraction of ERM and actin cortex from the PM. Anx A5 recruits the S100A4 protein to the surface of cells. Redox signaling changes the conformation of La, facilitating the delivery of oxidized La to the cell surface, where La directly interacts with Anx A5 and promotes fusion.

Several proteins involved in osteoclast fusion are united by their association with a common mechanistic motif: a transient exposure of PS on the surface of the cells (Figure 2). Anionic lipid PS is normally restricted to the inner leaflet of the PM of live cells. Osteoclast fusion and other cell–cell fusion processes are accompanied by and depend on PS appearance in the outer leaflet [45,49]. In osteoclast precursors, RANKL/RANK interactions trigger calcium signaling that activates lipid scramblases of the TMEM16 family, resulting in redistribution of PS from the inner to outer leaflet of the membrane [45,50]. RANKL also activates another phospholipid scramblase Xkr8, switched on by caspase 8 and downstream effector caspases [51]. PS delivered to the surface of the cells recruits PS-binding protein Annexin A5 (Anx A5). Note that in addition to proteins that directly mediate lipid scrambling, other proteins can promote scrambling by regulating the activity of scramblases. For instance, involvement of DC-STAMP in the PS externalization pathway is evidenced by the finding that DC-STAMP antibodies lower cell-surface exposure of PS [45].

#### Annexin A5

Anx A5 is a ubiquitous and abundant protein that belongs to a large family of structurally related proteins, whose common property is Ca^2+^-dependent binding of a conserved core domain to anionic phospholipids such as PS [52,53]. In the presence of Ca^2+^ and PS-containing lipid bilayer, Anx A5 forms homotrimers and can form a membrane-bound two-dimensional arrays generating negative (concave) curvature of the membrane [52]. Anx A5 is found in both the cytosol and the extracellular milieu and is implicated in numerous intra- and extracellular processes.

Extracellular Anx A5 involvement in the formation of multinucleated osteoclasts, and, specifically, in the cell fusion stage of osteoclastogenesis, has been demonstrated by several complementary experimental approaches. RANKL-induced osteoclastogenic differentiation dramatically increases Anx A5 presence at the surface of differentiating osteoclasts [45]. The application of recombinant Anx A5 promotes synchronized osteoclast fusion [50]. Conversely, knockdown or knockout of Anx A5 expression in human and murine osteoclasts, respectively, as well as the application of antibodies to Anx A5 and an Anx A5-derived peptide, inhibits the fusion and formation of multinucleated osteoclasts [45].

Application of Anx A5 to PS liposomes neither aggregates them nor induces lipid mixing [54]. This finding argues against the hypothesis that Anx A5, on its own, initiates lipid bilayer fusion. Furthermore, while Anx A5 promotes osteoclast fusion, it is not required for it. The rate of fusion in ex vivo cultures from Anx A5 knockout mice is only 50% lower than that for w.t. mice [45]. Anx A5 deficiency causes no gross skeletal abnormalities and no effect on bone mineral density [55]. The phenotypic manifestations of Anx A5 deficiency in the fusion stage can be moderated by the presence and contributions of other Annexins. Interestingly, fusion of murine osteoclasts depends, in addition to Anx A5, on another member of the Annexin family, Anx A1. While Anx A1 is also not essential for skeletal development, male Anx A1-knockout mice have a 25% higher bone mineral density than wildtype mice [56]. In contrast with murine osteoclasts, Anx A1-targeting reagents do not affect fusion of human osteoclasts [45]. However, we cannot exclude potential functional redundancy between different annexins or between Anxs and some other proteins. We still do not know whether Anx A5 contributions to fusion reflect the multifaceted effects of this protein on the lipid bilayer or its ability to recruit other proteins to PS-enriched patches of PMs, or some signaling triggered by Anx A5 binding to the cell surface.

At the surface of the cells, Anx A5 serves as a scaffold protein by interacting with other proteins [57–59]. In differentiating human osteoclasts, Anx A5 was found to bind S100A4 [45].

*S100A4*. The S100 family of calcium-binding proteins is involved in a wide range of cellular processes. Like other S100 proteins, S100A4 forms homooligomers of different sizes and complexes with different proteins, including Annexins [45,60]. While the best characterized functions of extracellular S100A4 are related to its pro-inflammatory and pro-metastatic activities [61], this protein is also implicated in bone remodeling. S100A4 deficiency in mice is associated with increased bone mass and characterized by osteoclasts having fewer nuclei and lower resorptive capacity [62]. The promotion of multinucleated osteoclast formation by S100A4, released by human periodontal ligament cells in inflammation and by bone metastatic breast cancer cells, has been suggested to contribute to bone destruction in periodontitis [63] and in breast cancer metastasis [64], respectively. In [45], we found that amounts of S100A4 associated with the surface of differentiating osteoclasts (human monocytes incubated with M-CSF and RANKL) were significantly higher than for the macrophage precursors (human monocytes incubated only with M-CSF). Antibodies to S100A4 inhibit synchronized osteoclast fusion. A synthetic peptide derived from the N-terminal domain of Anx A5 that inhibited osteoclast fusion was found to lower the amounts of cell surface S100A4 [45]. These findings suggest that the association of S100A4 with the cell surface depends on S100A4-Anx A5 binding and that this association is important for fusion. Interactions between the N-terminal region of another Annexin (Anx A2) and S100A4 have been reported to control membrane-membrane bridging by Anx-S100A4 complexes [60].

Besides S100A4, Anx A5 at the surface of differentiating osteoclasts was found to recruit extracellular La protein [65].

#### La protein

While evaluating RANKL-induced changes in protein expression during the formation of human osteoclasts in an *in vitro* model, we discovered a protein that was nearly absent from macrophage precursors but abundantly expressed in actively fusing osteoclasts [65]. La protein is an essential RNA-binding protein implicated in different aspects of RNA metabolism. In eukaryotic cells, this abundant and ubiquitous protein primarily exists as a phosphorylated nuclear phosphoprotein. In differentiating osteoclasts, La is dephosphorylated and cleaved by caspase 3 to remove the nuclear localization sequence, and then delivered to the surface of osteoclasts, where it directly binds to Anx A5. Lowering the amount of La by suppressing the steady-state level of its transcript, blocking its proteolytic processing, or inhibiting its activity at the cell surface with antibodies inhibits fusion. Conversely, increasing La’s steady-state concentration by either overexpression or application of recombinant protein promotes the cell fusion stage of osteoclastogenesis and increases resorptive capacity in osteoclasts. Interestingly, the most conserved and important for RNA recognition N-terminal half of the La sequence is dispensable for La’s role in osteoclast fusion [65].

Osteoclast formation, and specifically, osteoclast fusion, depends on RANKL-induced transient reactive oxygen species (ROS) signaling [66]. This signaling was found to control osteoclast multinucleation and function by triggering the change in intracellular La conformation and trafficking [66]. While reduced La species localizes within cell nuclei, oxidation of the critical cysteine residues in the C-terminal half of La facilitates dephosphorylation and proteolytic cleavage of La. Only the oxidized form of La traffics to the surface of osteoclasts, and only this form promotes osteoclast fusion.

In addition to direct binding of S100A4 and La, Anx A5 at the cell surface has recently been found to indirectly influence the actin cytoskeleton.

#### Anx A5 effects on actin cortex

As discussed above, fusion depends on membrane deformations, and the deformability of PM depends on its attachment to the underlying actin cortex (AC) mediated by Ezrin/Radixin/Moesin (ERM) proteins [67]. Osteoclastogenic differentiation is accompanied by and depends on weakening of AC-membrane attachment by the depletion of Moesin and Ezrin [35,50,68]. In addition to RANKL-induced down-regulation of ERM protein expression, detachment is mechanistically linked to the scramblase-dependent extracellular exposure of PS and Anx A5 binding [58]. ERM binding to the inner leaflet of PM depends on PS and another anionic lipid, phosphatidylinositol 4,5-bisphosphate (PI (4,5)P2), also highly enriched in this leaflet [69]. Scramblase-mediated lipid redistribution and extracellular Anx A5 binding to externalized anionic lipids lower PS and PI (4,5)P2 concentrations in the inner leaflet and, thus, promote local ERM- and AC detachment. This pathway is suggested to facilitate osteoclast fusion and other cell–cell fusions by promoting membrane deformations required for the formation of prefusion membrane contacts. Furthermore, AC detachments are suggested to influence fusion by changing membrane tension [50,68]. Theoretical analysis in [50] suggests that AC-PM detachment locally increases membrane tension and that this PS exposure- and Anx A5-dependent increase in tension promotes formation and expansion of a fusion pore.

The list of the proteins implicated in the regulation of the osteoclast multinucleation is far from complete [47,48]. In most cases, the specific contributions of identified protein players to the fusion process remain unclear. However, most of the proteins on these ever-growing lists likely act in controlling where, when, and which cells fuse. Fusion is a radical and, considering the suggested role of cell fusion in cancer, potentially risky change in cell physiology [37,70]. Making fusion contingent on the intersection of multiple intracellular signaling pathways, including Ca^2+^, redox, and lipid signaling, likely minimizes the risk of uncontrolled fusion.

Regardless of the specific contributions of different fusion-regulating proteins, these proteins can represent potential therapeutic targets. Some widely used approaches to treating bone loss diseases, such as antibodies to RANKL [71], are aimed at blocking very early stages of osteoclast formation. Targeting osteoclast fusion can be a more subtle way to attenuate bone resorption. While large, highly multinucleated osteoclasts are more efficient in resorbing bones [7] and, especially, performing fast and continuous ‘trench’ resorption [72,73], smaller and possibly even unfused mononucleated osteoclasts do resorb bones [42,74,75], mostly by less efficient pit-type mechanism [7]. Therefore, lowering the multinucleation reduces but preserves bone-resorbing activity and may be expected to less severely affect bone homeostasis than blocking pre-fusion stages of osteoclast differentiation. Furthermore, decreasing the size of osteoclasts rather than blocking their formation can keep some of the osteoclast-to-osteoblast signaling, by which osteoclasts, including smaller ones, regulate bone-forming activity of osteoblasts [76]. Advantages and limitations of different osteoclast fusion-targeting approaches and, more generally, the place of such approaches in the therapeutic arsenal remain to be clarified.

Below, we discuss some of the mechanisms by which identified fusion-associated proteins may directly contribute to the fusion of PMs.

### Proteins driving rearrangements of osteoclast membranes

Studies on fusion mediated by several well-characterized enveloped viruses and on SNARE-dependent intracellular fusion led to a generalized picture of ‘classical’ protein fusogens, as homo- or hetero-oligomeric complexes anchored in one of the membranes or distributed between two membranes (reviewed in [37]). At the time of fusion, changes in oligomerization state and/or conformation of the proteins from their metastable higher energy conformations toward their lower energy post-fusion forms pull together their anchors (transmembrane domains and fusion peptides inserted into the membrane). This jackknifing of the tall and rigid protein complex brings together two membranes, while interactions between the amphiphilic regions of the proteins and the membranes reduce the energy of fusion intermediates. In this case, a single protein or protein complex drives the membrane fusion process. Many proteins with tall ectodomains have been suggested to drive fusion by this elegant mechanism [77,78].

Cell–cell fusion may be driven by proteins structurally similar to classical fusogens. Syncytin-dependent trophoblast fusion in mammals, EFF-1 and AFF-1 dependent fusion of hypodermis and anchor cells in *C. elegans*, and HAP2-dependent fusion in algae and plants are prime examples of classical protein fusogens in cell–cell fusion. However, in the case of myoblast fusion, where two muscle-specific proteins, Myomaker and Myomerger (also known as Myomixer and Minion), essential for the formation of multinucleated muscle cells [79], drive distinct stages of the fusion process—hemifusion and pore formation—independently of each other, neither of these proteins resembles ‘classical’ protein fusogens.

In the case of osteoclast fusion, proteins essential for fusion and capable of inducing fusion when overexpressed in a heterologous system have yet to be identified. The human endogenous retrovirus envelope glycoprotein Syncytin 1 (Syn-1) is a good candidate for a protein directly mediating fusion of osteoclast PMs. Fusogenic activity of this protein triggered by its interactions with its ASCT1/2 receptors is well established in the context of trophoblast fusion and cancer cells, in various heterologous cell types, and in the fusion of pseudoviruses. The involvement of Syn-1 in osteoclast fusion was first suggested by Kent Soe and co-authors [38,80]. Finding that antibodies to Syn-1 and a peptide specifically blocking fusogenic restructuring of this protein from pre-fusion to post-fusion conformation inhibited synchronized fusion of human osteoclasts confirmed the functional role of cell surface Syn-1 in fusion [45]. The involvement of Syncytins in osteoclast fusion is further supported by experiments on murine osteoclasts [81]. While Syn-1 and another endogenous retroviral Syncytin (Syn-2), also essential in placentogenesis, are human proteins, Syn-A and Syn-B are their counterparts in mice. Syn-B knockout lowers the number of multinucleated osteoclasts in murine ex vivo cultures. Importantly, suppressing Syncytins in osteoclasts with antibodies, peptide inhibitors, and even, in the case of Syn-B, by permanently inactivating its expression, does not abolish fusion but only lowers its efficiency by ~50%. Together, while there is substantial evidence supporting the hypothesis that osteoclasts utilize Syncytins as protein fusogens, a considerable level of redundancy exists in their function. Furthermore, RANKL-induced osteoclastogenic differentiation is not accompanied by up-regulation of Syn-1 [45], its receptor ASCT2 [80], or Syn-B [81], indicating that the function of Syncytins in osteoclast fusion is controlled not only by the receptor interactions.

**Figure 3:**
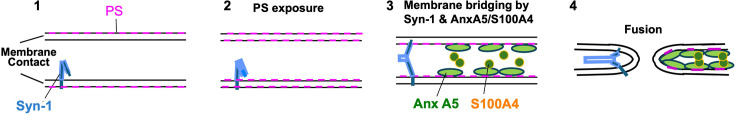
Hypothetical mechanism of the membrane fusion stage in osteoclastogenesis. Downstream of cell surface exposure of PS (shown as a pink line), Syn-1, Anx A5, and S100A4 (shown as blue, green, and orange shapes, respectively) collaborate in pulling the membranes together and initiating fusion. Modified from [45].

In [45], we suggested that while at high enough concentrations characteristic for trophoblasts, Syn-1 fuses membranes on its own, at lower levels of Syn-1 expression, which are characteristic for osteoclasts, fusogenic activity of Syn-1 is supported by the effects of cell surface Anx A5 and S100A4 (Figure 3). Upon binding to its receptors, Syn-1 trimer undergoes conformational changes that allow its fusion peptides to insert into the target membrane and to form a short-living extended conformation of the protein with exposed N-terminal heptad repeats and the C-terminal helices. If the number of extended conformations of Syn-1 is insufficient to drive close approach and fusion of the membranes, proteins transit into inactivated state conformations. On the other hand, the formation of the extended conformation of the Syn-1 facilitates an additional membrane-bridging mechanism provided by Anx A5-S100A4 complexes assembled at the externalized PS. This Anx-based bridging mechanism, along with the final restructuring of Syn-1, in which the N-terminal and C-terminal domains form the lowest energy hairpin conformation, pulls together and fuses the membranes. In addition to bridging, cell surface Anx A5 can potentially contribute to bilayer fusion by generating negative curvature of the opposing lipid monolayers of the membranes [52]. This highly speculative hypothesis does not explain the regulatory effects of La-Anx A5 binding.

The search for proteins that drive osteoclast fusion from adhesion contact to opening of a fusion pore can be hindered by overestimating the demands of the job. The requirements for a protein or protein complex that mediates unhurried cell–cell fusion, which develops over days, can be much less stringent, if fusion is initiated in thin plasma membrane protrusions [82]. The membrane of thin protrusions is mechanically stressed and may be depleted of proteins, providing an easier contact between lipid bilayers. Indeed, a very short membrane-disrupting ectodomain is all that is needed for fusogenic activity of FAST proteins, provided these proteins are propelled forward by actin polymerization-driven thin filopodia. Considering that osteoclast fusion indeed involves the formation of outward-going membrane protrusions [33,83–85], classical fusogens may not be required, placing all important decisions to fuse or not to fuse at the pre-fusion stages. As discussed above, fusion competence develops as a convergence of several regulatory mechanisms, including RANKL/RANK-, redox-, calcium-, lipid-, and actin cytoskeleton- signaling pathways.

## Concluding remarks

The molecular mechanisms that regulate the bone-resorbing activity of osteoclasts by controlling their size remain to be fully clarified. Taken together, current evidence suggests that osteoclast fusion is orchestrated by a complex interplay of multiple proteins, which is temporally and spatially co-ordinated through calcium signaling, PS externalization, and actin cytoskeleton remodeling. However, significant gaps in our knowledge remain, particularly regarding the precise molecular pathways by which these proteins interact and drive membrane merger and fusion pore formation. A better understanding of these pathways and specific role of different proteins will not only advance our understanding of osteoclast biology and mechanisms by which proteins initiate and drive diverse fusion processes but may also provide novel therapeutic targets for skeletal diseases.

**Perspectives Section**

- Highlight the importance of the field: The fusion of osteoclasts regulates the bone-resorbing activity of these multinucleated cells during lifelong remodeling of the bones and in skeletal diseases.
- Summary of the current thinking: The all-important decisions to fuse or not to fuse are controlled by multiple intracellular signaling pathways, including Ca^2+^-, redox-, and lipid-signaling, as well as actin cytoskeleton rearrangements. The cell fusion step in osteoclastogenesis depends on the phosphatidylserine-regulated activity of several proteins; however, the molecular mechanisms of this multiprotein machinery remain elusive.
- Comment on future directions: We expect future work to clarify (1) whether there are proteins expressed in osteoclasts at the time of their fusion that are both essential for fusion and capable of inducing fusion when overexpressed in a heterologous system; and (2) elucidate the mechanisms by which DC-STAMP, Annexin A5, and La protein at the surface of osteoclasts regulate cell fusion.

## Open Access

This article has been published open access under our Subscribe to Open programme, made possible through the support of our subscribing institutions, learn more here: https://portlandpress.com/pages/open_access_options_and_prices#conditional

## Abbreviations

Anx A5: Annexin A5
DC-STAMP: dendritic cell-specific transmembrane protein
ERM: Ezrin/Radixin/Moesin
FAST: fusion-associated small transmembrane
LPC: lysophosphatidylcholine
M-CSF: macrophage colony-stimulating factor
M-CSF: macrophage colony-stimulating factor
OC-STAMP: osteoclast stimulatory transmembrane protein
PC: phosphatidylcholine
PI(4,5)P2: phosphatidylinositol 4,5-bisphosphate
PMs: plasma membranes
PS: phosphatidylserine
RANKL: receptor activator for NF-κB (nuclear factor κB) ligand
Syn-1: Syncytin 1
